# Hepatic Artery Thrombosis After Orthotopic Liver Transplant: A 20-Year Monocentric Series

**DOI:** 10.3390/jcm14134804

**Published:** 2025-07-07

**Authors:** Vincenzo Tondolo, Gianluca Rizzo, Giovanni Pacini, Luca Emanuele Amodio, Federica Marzi, Giada Livadoti, Giuseppe Quero, Fausto Zamboni

**Affiliations:** 1 UOC Chirurgia Digestiva e del Colon-Retto—Ospedale Isola Tiberina Gemelli Isola, 00186 Rome, Italy; vincenzo.tondolo@fbf-isola.it (V.T.); lucaemanueleamodio@virgilio.it (L.E.A.); federicamarzi9@gmail.com (F.M.); giada.livadoti@fbf-isola.it (G.L.); 2 Dipartimento di Medicina e Chirurgia Traslazionale, Università Cattolica del Sacro Cuore, 00168 Rome, Italy; giuseppe.quero@policlinicogemelli.it; 3 Centro Trapianti—Azienda Ospedaliera «G. Brotzu», 09047 Cagliari, Italy; gio.pacini93@gmail.com (G.P.); fausto.zamboni@aob.it (F.Z.); 4 UOC Chirurgia Digestiva—Fondazione Policlinico Universitario Agostino Gemelli IRCCS, 00168 Rome, Italy

**Keywords:** hepatic artery thrombosis (HAT), early HAT, late HAT, orthotopic liver transplantation (OLT), orthotopic liver re-transplantation (Re-OLT)

## Abstract

**Background/Objectives**: Hepatic artery thrombosis (HAT) is a serious vascular complication in patients undergoing orthotopic liver transplantation (OLT). It is associated with a high risk of graft loss, re-transplantation (re-OLT), and mortality. This study aimed to evaluate the incidence and management of HAT, analyzing potential risk factors. The secondary objectives included quantifying 90-day postoperative morbidity and mortality rates. **Methods**: In this retrospective, observational, single-center study, data from liver transplant donors and recipients who underwent OLT between 2004 and 2024 were analyzed. HAT was classified as early (e-HAT, ≤30 days) or late (l-HAT, >30 days). Univariate statistical analysis was performed to identify the risk factors associated with HAT occurrence. Multivariate analysis was not performed due to the small number of HAT events, which would increase the risk of model overfitting. **Results**: In the 20 year study period, a total of 532 OLTs were performed, including 37 re-OLTs. The rates of major morbidity, reoperation, and mortality within 90 days were 44.5%, 22.3%, and 7.1%, respectively. HAT occurred in 2.4% of cases (e-HAT: 1.6%; l-HAT: 0.7%). Among e-HAT cases, 66.6% were asymptomatic and identified through routine postoperative Doppler ultrasound. All e-HAT cases were surgically treated, with a re-OLT rate of 33.3%. Three l-HAT cases required re-OLT. Overall, the HAT-related mortality and re-OLT rates were 7.6% and 46.1%, respectively. At a follow-up of 86 months, the rate of graft loss was 9.2%, and the rate of post-OLT survival was 77%. Patients who developed HAT had a higher donor-to-recipient body weight ratio and longer warm ischemia times (WITs). Additionally, patients undergoing re-OLT had a higher risk of developing HAT. **Conclusions**: Although the incidence of HAT is low, its clinical consequences are severe. Early Doppler ultrasound surveillance is crucial for detecting e-HAT and preventing graft loss. A high donor-to-recipient body weight ratio, a prolonged warm ischemia time, and re-OLT seem to be associated with a high risk of HAT.

## 1. Introduction

Orthotopic liver transplantation (OLT) is the standard surgical treatment for end-stage liver failure resulting from acute or chronic liver diseases [1]. It also serves as an effective surgical option for certain primary and secondary liver malignancies, such as hepatocellular carcinoma and neuroendocrine tumor metastases. Moreover, within research protocols (particularly in Italy), OLT is increasingly employed for the treatment of cholangiocarcinoma and colorectal cancer metastases [1].

OLT is a major surgical procedure associated with a substantial risk of postoperative morbidity and mortality, which varies based on the recipient’s risk profile [2,3,4,5].

Complications involving the hepatic artery are particularly dangerous, as they can lead to graft ischemia, resulting in graft loss and the need for re-transplantation (re-OLT), both of which carry a high mortality risk [6]. The incidence of these complications varies widely, ranging from 2% to 20%, depending on the type (thrombosis or stenosis) and timing (early or late) of the arterial complication in relation to the time of OLT [7].

Hepatic artery thrombosis (HAT) is considered to be the most common vascular complication involving the hepatic artery, with an incidence in adult patients ranging from 1.9% to 9% [8,9,10]. It is also the most severe, as it is associated with a high risk of major morbidity—including abscesses, cholangitis, and hepatic necrosis—re-OLT, and a mortality rate of up to 50% [8,11]. When HAT occurs within four weeks of OLT (early HAT), its clinical consequences are more severe and may include acute graft failure, sepsis, liver abscesses, or biliary complications, such as leaks or strictures [7]. In contrast, the clinical presentation of late HAT is more variable and may include cholangitis (with or without strictures or abscesses), bile leaks, or abnormal liver function tests [7].

The treatment of early HAT often requires surgical intervention, including re-OLT when feasible, depending on both graft availability and patient condition [12]. Early diagnosis is essential to enable timely, targeted interventions that preserve graft function, reduce the need for re-OLT, and lower HAT-related mortality. Urgent revascularization—either by thrombectomy or thrombectomy combined with the revision of the arterial anastomosis—has proven successful in some early HAT cases. Early detection is made possible by rigorous surveillance using Doppler ultrasonography in the immediate postoperative period [13,14,15].

The etiology of HAT remains controversial and is often unclear. While surgical techniques have traditionally been considered the most critical risk factor [12], several others have been identified, including graft preservation quality, ischemia–reperfusion injury, immunological factors, coagulation disorders, infections, pediatric transplantation, elderly donors, re-OLT, the use of arterial conduits, prolonged operative times, and low recipient body weight [16,17,18,19].

Few studies have reported long-term, single-center experiences of HAT and analyzed the potential risk factors in a setting of strictly standardized surgical technique and postoperative care. Therefore, the primary aim of this study was to evaluate the incidence of both early and late HAT after OLT, its management, and its impact on short- and long-term post-transplant outcomes. Additionally, the study sought to identify potential risk factors associated with HAT occurrence over a 20-year period at a single center. In particular, it was hypothesized that specific intraoperative and donor–recipient variables may be significantly associated with the development of HAT following OLT. The secondary aims included a quantitative and qualitative analysis of major postoperative morbidity (≥grade 3 according to the Clavien–Dindo classification) and mortality within 90 days of OLT.

## 2. Materials and Methods

All adult patients who underwent whole-organ orthotopic liver transplantation (OLT) at “G. Brotzu” Hospital in Cagliari between 1 March 2004 and 1 March 2024 were included in a prospective database and retrospectively analyzed. For each case, donor and recipient characteristics, intraoperative data, and both short- and long-term postoperative outcomes were collected. A standardized surgical approach to OLT has consistently been used at our institution.

### 2.1. Organ Procurement and Back-Table Procedure

All surgeons followed a standardized surgical technique. Organ procurement was performed using rapid arterial perfusion via the abdominal aorta with University of Wisconsin cold preservation solution, followed by en bloc removal of the liver, pancreas, and spleen. Hepatic pedicle dissection was conducted on the back table. In cases of arterial anomalies in the graft, aberrant vessels (accessory or replaced arteries) were reimplanted. All anastomoses were performed with continuous 7-0 polypropylene sutures, using microsurgical techniques and instruments under 3.5× or 4.5× magnification.

### 2.2. Operative Details

Reconstruction of the caval vein was always performed using the classical piggyback technique. Allograft reperfusion was achieved via portal vein anastomosis, performed with continuous 5-0 polypropylene sutures. In cases of inadequate venous outflow from the terminal caval anastomosis, a rescue side-to-side cavo-cavostomy was performed to create a new opening between the two caval veins.

Arterial anastomosis was carried out end-to-end between the donor’s proper hepatic artery (dPHA) or common hepatic artery (dCHA) and the recipient’s proper hepatic artery (rPHA), common hepatic artery (rCHA), or right hepatic artery (rRHA). The goal was to create a short arterial segment to prevent torsion or kinking. The arterial knot was intentionally left slightly loose to allow for micro growth factor activity, promoting optimal anastomotic distension after reperfusion.

When standard reconstruction was not feasible due to caliber discrepancies, vessel wall abnormalities, or technical difficulties, alternative revascularization techniques were employed, such as back-table reconstructions; jump grafts using iliac artery conduits from the donor; arterial flow optimization via ligation of the gastroduodenal artery, splenic artery, or both; multiple arterial anastomoses.

Intraoperative Doppler ultrasound was systematically used to confirm adequate arterial flow, repeated after abdominal closure if necessary.

Biliary reconstruction was performed via hepatic–choledochal anastomosis with a Kehr tube, or via hepaticojejunal anastomosis using 5-0 or 6-0 polydioxanone sutures on a Roux-en-Y loop.

### 2.3. Postoperative Management

All patients received immunosuppressive therapy based on tacrolimus, combined with mycophenolic acid and basiliximab for induction. In HCV-related cases, induction was instead achieved using a low-dose steroid bolus (20 mg) with rapid tapering. Antiplatelet prophylaxis with aspirin (ASA) at 100 mg/day was administered if the platelet count exceeded 1,000,000/mm^3^. When feasible, immunosuppression was switched to an everolimus-based regimen no earlier than six months post-transplant.

During hospitalization, patients underwent daily monitoring with laboratory tests, including complete blood count, liver function tests, coagulation parameters, and inflammatory markers. Twice-weekly screening for CMV infection (via DNA testing) was performed, along with serologic testing for other pathogens (herpes, toxoplasma, varicella, etc.). CMV-negative recipients receiving a graft from a CMV-positive donor received valganciclovir prophylaxis; any patient with a positive CMV DNA test (>500 copies) was treated with valganciclovir for 21 days, continued until three consecutive negative results were obtained.

Liver Doppler ultrasonography was routinely performed daily from postoperative day 1 to day 10, and later if clinically indicated. The frequency has been twice daily in the presence of normal findings after standard arterial reconstruction [20]. In patients with complex arterial reconstructions or high-risk Doppler findings—such as “tardus parvus” waveform, absent diastolic flow, or elevated intrahepatic resistance indexes—more frequent assessments (up to 4 times per day) were conducted. If HAT was suspected, contrast-enhanced triple-phase abdominal CT and, when necessary, angiography were used for confirmation. In cases of increased cytolysis or cholestasis markers without vascular abnormalities, liver biopsy was performed to confirm rejection. Rejection was treated according to severity, ranging from the adjustment of immunosuppressive therapy to high-dose steroid pulses (hydrocortisone 1 g IV for three days).

After discharge, patients were followed regularly by transplant surgeons and hepatologists, with liver Doppler ultrasonography routinely performed, including after discharge, for 10 days after OLT. During the first year post-OLT, follow-up visits occurred for 1 month and every 3 months thereafter and included clinical evaluation, lab tests, and vascular Doppler ultrasound. From the second to fifth year post-OLT, Doppler ultrasound was performed biannually. In patients transplanted for HCC, contrast-enhanced CT scans were performed at 6 months, at 1 year, and annually through year 3.

### 2.4. Data Analysis

All postoperative complications occurring during hospitalization or within 90 days of OLT were recorded as short-term complications and classified according to the Clavien–Dindo system [21]. Complications graded ≥3 were categorized as major. The following outcomes were also recorded:

- Length of post-OLT hospital stay (mean±SD);
- Reoperations within 90 days;
- Re-OLT within 90 days;
- 90-day post-OLT mortality;
- Duration of post-OLT follow-up (from transplant to last outpatient visit);
- Overall post-OLT survival (from transplant to last follow-up or death);
- Overall re-OLT rate;
- Graft loss rate (defined as patient death or need for re-OLT).

HAT occurrence was recorded and classified as early (within 30 days of OLT) or late (after 30 days). The following HAT-related outcomes were also documented:

- Postoperative day of onset;
- Presence of symptoms (yes/no);
- Type of treatment (medical or surgical);
- HAT-related mortality (short-term within 90 days and long-term).

All patients provided written informed consent for the surgical procedure and for the use of their clinical data for research purposes, including data processing. The database used for this study was coded to ensure confidentiality. Due to its retrospective design and the use of an anonymous database, this study does not require approval from the local Ethics Committee. Moreover, all patients who underwent liver transplantation provided, in addition to informed consent for the surgical procedure, consent for the use of their clinical data for scientific purposes. Therefore, they also consented to the processing of their personal data.

### 2.5. Statistical Analysis

Statistical analysis was performed to assess predictive and protective factors associated with HAT. Table 1 lists all variables included in the analysis. For each variable, univariate statistical analysis was conducted. Univariate analyses were performed using chi-square or Fisher’s exact test for categorical variables and Student’s *t*-test or Mann–Whitney U test for continuous variables, depending on data distribution. A *p*-value < 0.05 was considered statistically significant. Multivariate analysis was excluded due to the small number of HAT events (*n* = 13), which would render the analysis statistically unreliable due to overfitting risk.

## 3. Results

Between March 2004 and February 2024, 532 adult whole-organ OLTs were performed, including 37 re-OLTs. Table 2, Table 3, Table 4, Table 5 and Table 6 summarize the data on donors, recipients, and perioperative factors. All transplants were performed exclusively by two surgeons (FZ and VT), consistently using the same standardized and well-established technique over the years.

### 3.1. Donor Characteristics (Table 2)

The median donor age was 57.5 years (IQR: 42–72 years; range: 15–79 years), with a slight predominance of male donors (285; 53.0%). The median BMI was 24.97 kg/m^2^ (IQR: 23.04–27.14 kg/m^2^). The most common cause of death was cerebral hemorrhage (304 donors, 57.1%), while cardiac arrest prior to organ procurement occurred in 76 donors (14.2%), and hypotensive episodes in 28.3% of cases. Blood transfusions were required in 115 donors (21.6%). The median ICU stay was 3 days (IQR: 2–5 days). No DCD (donation after circulatory death) or living donations were performed during the study period. Cytomegalovirus (CMV) IgG positivity was found in 82.8% of donors, and 18.7% were anti-HBc Ag-positive. Graft steatosis was observed in 245 donors (46.0%), with macro-vesicular characteristics in 181 donors (34.0%). The median graft weight was 1378 g (IQR: 1166–1581 g), and 28.1% of cases had a positive culture result from organ preservation fluid. The median donor-to-recipient body weight ratio (BWR) was 1 (IQR: 0.87–1.18).

### 3.2. Recipient Characteristics (Table 3)

The median recipient age was 54 years (IQR: 48–60 years), with a marked male predominance (440; 82%). The median BMI was 23.9 kg/m^2^ (IQR: 22.6–26.8 kg/m^2^). CMV seropositivity was found in 89.4% of recipients, and 8% received a liver from a CMV-positive donor. The most frequent indications for liver transplantation (Table 4) in our series were alcohol-related liver cirrhosis (250 cases; 46.9%) and hepatocellular carcinoma (202 cases; 37.9%). The median biological model for end-stage liver disease (MELD) score was 16 (IQR: 11–22). Among the 202 patients transplanted for HCC, 80 (39.6%) had undergone pre-OLT treatment with chemoembolization (TACE), trans-arterial radioembolization (TARE), or hepatic resection.

### 3.3. Intraoperative Factors (Table 5)

A total of 532 OLTs were performed, including 37 re-OLTs (6.9%) and 8 (0.1%) combined with kidney transplantation. The median cold ischemia time (CIT) was 400 min (IQR: 350.5–462 min), and the median warm ischemia time (WIT) was 30 min (IQR: 25–37).

A standard arterial anastomosis was performed in 88.4% of cases, typically between the donor’s proper hepatic artery (dPHA) or common hepatic artery (dCHA) and the recipient’s common hepatic artery (rCHA), proper hepatic artery (rPHA), or large right hepatic artery (rRHA). Detailed arterial anastomosis types are shown in Table 6. An arterial conduit was required in four cases (0.7%). Vascular graft anomalies occurred in 149 cases (28.0%), requiring back-table reconstruction. Gastroduodenal artery (GDA) and splenic artery (SA) ligation was performed in 152 cases (28.6%), GDA ligation alone in 135 cases (25.3%), and SA ligation alone in 13 cases (2.4%). More than one vascular anastomosis was performed in 14 OLTs (2.6%).

Biliary reconstruction was achieved via choledochocholedochal or hepaticocholedochal anastomosis in 290 cases (54.5%), while the remaining cases underwent bilioenteric anastomosis.

### 3.4. Postoperative Outcomes (Table 7)

The rate of major morbidity (Clavien–Dindo ≥ 3) within 90 days post-OLT was 44.5%, with surgical reintervention required in 119 cases (22.3%), including 37 re-OLTs (6.9%). A biopsy-proven rejection occurred in 48 patients (9.02%). The median postoperative hospital stay was 15 days (IQR: 7–39 days). The 90-day post-OLT mortality rate was 7.1%.

**Figure 1:**
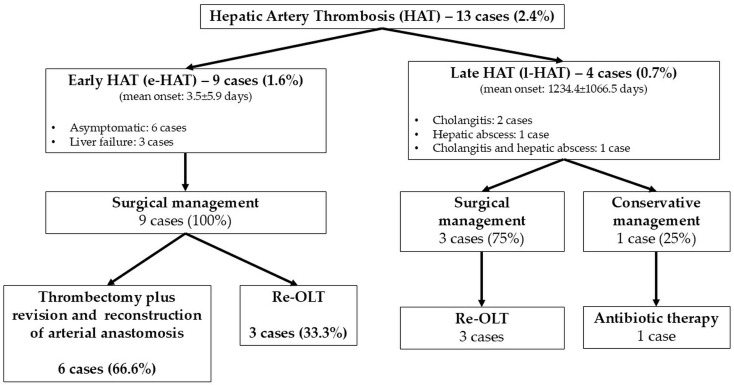
Incidence and management of HATs.

In our series, hepatic artery thrombosis (HAT) occurred in 13 cases (2.4%), with early HAT (e-HAT) in 9 cases (1.6%) and late HAT (l-HAT) in 4 cases (0.7%). The mean onset of e-HAT was 3.5 ± 5.9 days postoperatively. In six cases (66.6%), patients were asymptomatic, and e-HAT was detected via Doppler ultrasound. In the remaining three cases (33.3%), liver failure was observed. All cases of e-HAT required surgical treatment: in six patients (66.6%), thrombectomy with revision and reconstruction of the arterial anastomosis was performed, while re-OLT was necessary in three cases (33.3%), with one patient dying postoperatively (Figure 1).

For l-HAT, the mean onset was 1234.4 ± 1066.5 days post-OLT. Symptoms included cholangitis in two patients (50%), hepatic abscess in one patient (25%), and both conditions in one patient (25%). In three cases (75.0%), surgical treatment with re-OLT was performed; in only one case (25.0%), a conservative approach with intravenous antibiotic therapy was successful in avoiding re-OLT (Figure 1).

The overall HAT-related mortality and re-OLT rates were 7.6% and 46.1%, respectively. None of the HAT patients had a biopsy-proven rejection.

**Table 7:** Post-OLT outcomes.

Outcome	*n* (%)
Post-OLT mortality within 90 days	7.1%
Post-OLT morbidity within 90 days	420 (78.9%)
Grade ≥3 morbidity according to Clavien–Dindo class	237 (44.5%)
Reoperation	119 (22.3%)
Hemostasis revision	41 (7.7%)
Abdominal cavity lavage	13 (2.4%)
Creation of bilio-digestive anastomosis	31 (5.8%)
Reconstruction of the hepatic artery	6 (1.1%)
Cavo-cavostomy	2 (0.3%)
Overall re-OLT rate	37 (6.9%)
Re-OLT for HAT	6 (1.1%)
Re-OLT for other causes	31 (5.8%)
Median post-OLT hospital stay (IQR)	15 (7–39) days
HAT rate	13 (2.4%)
Early HAT	9 (1.6%)
Late HAT	4 (0.7%)
HAT management (on 13 cases)	
Re-OLT	6 (46.0%)
Reconstruction of arterial anastomosis	6 (46.0%)
Medical treatment	1 (7.6%)
Overall HAT-related reoperation rate	12 (92.3%)
HAT-related mortality	1 (7.6%)

The statistical analysis (Table 8) identified a high body weight ratio (1.22 in HAT vs. 1.03 in non-HAT; *p* = 0.006), the need for re-OLT due to graft failure (15.4% in HAT vs. 2.7% in non-HAT; *p* = 0.008), prolonged WIT (40.9 ± 17.1 min in HAT vs. 31.9 ± 0.9 min in non-HAT; *p* = 0.004), and the need for rescue cavo-cavostomy (15.4% in HAT vs. 1.9% in non-HAT; *p* < 0.001) as risk factors for HAT after OLT.

The overall mean follow-up period was 86 months. During this time, a graft loss occurred in 9.2% of cases. The overall post-OLT survival was 77%.

## 4. Discussion

Hepatic artery thrombosis (HAT) is a rare but serious vascular complication following orthotopic liver transplantation (OLT), with potentially life-threatening consequences. In our 20-year series, the overall incidence of HAT was 2.4%, with early HAT (e-HAT) at 1.6% and late HAT (l-HAT) at 0.7%. These rates are consistent with those reported in the literature. For instance, an Italian series by Pinna et al. had an overall HAT incidence of 5.4% (e-HAT 3.6%) [6]; Yi et al. found an incidence of 2.7% (e-HAT 1.9%) [22], while a 10-year Hispanic cohort had an e-HAT rate of 5.33% [23].

The relatively low HAT rate in our series—particularly the 1.6% e-HAT rate—may represent a favorable outcome, likely attributable to three key factors: (1) the consistent use of microsurgical techniques under 3.5× or 4.5× magnification; (2) the creation of short arterial segments to avoid kinking and torsion; and (3) the use of a micro growth factor within the suture knot to optimize anastomotic distension. Studies have demonstrated that the use of microsurgical arterial reconstruction significantly reduces HAT incidence from 2 to 24.1% (without microsurgery) to 0–2.1% (with microsurgery) [24,25,26,27].

The severe consequences of e-HAT, frequently resulting in graft loss, often necessitate aggressive surgical interventions to preserve the graft. Despite such efforts, many cases still require re-OLT [28,29]. In contrast, re-OLT in l-HAT cases is often driven by complications of ischemic cholangiopathy [29,30]. In our series, all patients with e-HAT underwent surgical revision, with re-anastomosis performed in 66% and re-OLT required in 33% of cases. No interventional radiological procedures (e.g., stenting or dilatation) were employed in order to preserve arterial anatomy for possible re-OLT. Among l-HAT cases, 75% required re-OLT. Our overall re-OLT rate for HAT was 46%, comparable to previous reports. In a German cohort, the re-OLT rate was 46.7%, with 40% of HAT cases managed conservatively [31]. That study also reported a 16% mortality rate following re-OLT, identical to our observed rate. In contrast, a Chinese cohort reported a higher overall HAT-related mortality rate of 42.9% [32], while a large study from the University of Birmingham reported a rate of 30.4% [29].

Timely diagnosis of e-HAT is critical to improving clinical outcomes. Studies have shown significantly better graft survival when e-HAT is detected asymptomatically via Doppler ultrasound in the immediate postoperative period [33,34]. In our cohort, all patients underwent standardized Doppler ultrasound monitoring from postoperative day 1 to day 5, with more frequent assessments for high-risk cases. This protocol enabled early detection and led to a 66% graft salvage rate among e-HAT patients.

Among the identified risk factors for HAT, the donor-to-recipient body weight ratio emerged as particularly significant: higher ratios were associated with increased HAT risk. The donor-to-recipient body weight ratio reflects a mismatch in arterial caliber and flow dynamics between the graft and the recipient. A higher body weight ratio implies that the donor liver is disproportionately larger relative to the recipient’s body. This discrepancy can result in altered hemodynamic conditions within the hepatic artery, such as increased shear stress and turbulence at the anastomosis site due to size mismatches; a higher flow demand from the graft compared to the recipient’s arterial capacity, potentially leading to endothelial damage. Greater susceptibility to arterial kinking or torsion, especially when the donor artery is longer or bulkier, potentially increases the chance of flow disruption and thrombosis. This finding is consistent with those reported in the previous literature. For example, Yang et al. found that a ratio >1.15 was significantly associated with e-HAT [22], and Oh et al. observed increased risk when the ratio exceeded 1.25 [35]. In our study, HAT patients had a mean warm ischemia time (WIT) of 40.9 min. Prolonged WIT has also been shown to increase HAT risk. Extended WIT exacerbates ischemia–reperfusion injury, which triggers a cascade of pro-inflammatory and pro-thrombotic responses: reperfusion of ischemic tissue leads to oxidative stress and endothelial dysfunction, reducing nitric oxide bioavailability and promoting vasoconstriction and platelet aggregation; ischemia–reperfusion injury also activates the coagulation cascade, increasing the likelihood of microthrombi formation within the hepatic artery; the pro-inflammatory milieu can lead to vasospasm, narrowing the arterial lumen and further compromising blood flow. Moreover, in complex cases, surgical time may be prolonged, and WIT may increase as a secondary consequence, thereby compounding the risk. In the series by Veras Pinto et al., a WIT >35 min significantly increased HAT incidence (reported rate: 2.8%) [36]. Warner et al. found that each additional 10 min delay in reperfusion increased the risk of e-HAT by 27% [37]. Complex surgical scenarios, such as unexpected portal vein thrombosis, bleeding from the caval anastomosis, and longer operative times, may contribute to a prolonged WIT and elevated HAT risk [36]. Yang et al. also reported that arterial anastomosis times exceeding 80 min and total OLT durations over 10 h were associated with significantly higher HAT incidence [22].

The need for re-OLT due to graft failure and the use of rescue cavo-cavostomy were two additional risk factors strongly correlated with HAT in our series. Rescue cavo-cavostomy, performed in only 2.4% of cases, is an emergency procedure used in the presence of poor hepatic venous outflow and is associated with complex surgical conditions and delayed arterial reconstruction. Similarly, Silva et al. found an increased risk of e-HAT following re-OLT, likely due to technical challenges such as difficult arterial reconstructions, limited implantation sites, and the use of aorto-iliac conduits, each of which has been linked to elevated HAT risk [38].

The role of pharmacologic prophylaxis for HAT prevention remains a topic of ongoing debate, particularly concerning the choice between antiplatelet agents, such as aspirin and systemic anticoagulation. At our institution, in line with findings from Kirchner et al. [37], aspirin prophylaxis (100 mg/day) is used in selected patients with platelet counts exceeding 1,000,000/mm^3^, as it may reduce HAT risk. In our series, although we did not observe major hemorrhagic complications related to ASA use, the low overall incidence of HAT in our cohort may suggest a protective effect, consistent with prior findings from Kirchner et al. [39] and Feldman et al. [19]. However, the literature presents a more nuanced view. Several centers have adopted anticoagulation regimens using low-molecular-weight heparin (LMWH) or unfractionated heparin in the early postoperative phase, often followed by oral anticoagulants, such as warfarin. Proponents argue that anticoagulation more effectively mitigates the procoagulant state seen after liver transplantation, especially in recipients with portal hypertension, inherited thrombophilia, or intraoperative thrombosis. Studies in pediatric populations and high-risk adult recipients have demonstrated lower HAT rates with anticoagulants, albeit with a significant bleeding risk, particularly in the early post-OLT period when coagulopathy is common [11,19]. By contrast, antiplatelet agents, such as aspirin, are generally considered safer, easier to administer, and less likely to cause significant bleeding. Their mechanism of action, targeting platelet aggregation, addresses the early phase of thrombus formation at the anastomotic site. A recent expert consensus [39] concluded that in low-risk adult patients undergoing OLT, aspirin monotherapy may be sufficient for HAT prophylaxis, particularly when meticulous surgical technique and Doppler surveillance are employed [39].

In our series, no interventional radiological procedures, such as intra-arterial thrombolysis, stenting, or balloon angioplasty, were employed for the management of HAT. This was an intentional institutional choice. Our center prioritized surgical revision in cases of early HAT because it is always considered a technical problem of anastomotic stenosis or vessel kinking. Reoperation guarantees direct visualization of the problem and correction of technical defects. Furthermore, preservation of native vascular anatomy, non-use of intra-arterial catheters and/or stents for potential re-transplantation was considered essential, particularly in hemodynamically unstable patients or those with severe graft dysfunction. While some centers have reported favorable outcomes with endovascular interventions, particularly in late HAT or in patients unfit for surgery [28,29], the lack of standardized protocols and limited experience with these techniques at our institution has led us to prefer surgical approaches. We acknowledge this as a limitation of our study and recognize that a multidisciplinary approach, including early consultation with interventional radiology teams, may offer additional therapeutic options, especially in select late HAT cases or in asymptomatic patients with partial occlusion.

In our series, post-OLT major morbidity and 90-day mortality rates were 44.5% and 7.1%, respectively. Reintervention was necessary in 22.3% of cases, and 6.9% underwent re-OLT. The mean hospital stay was 15.6 days. These outcomes are consistent with the existing literature, which reports complication rates of 40–60% and graft loss rates of 30–40% [5,40].

This study has several limitations. The most significant is the long 20-year study period and the small number of HAT cases, which limits the statistical power of our analyses and makes multivariate analysis unreliable due to the risk of overfitting. However, the low incidence of HAT reflects the rarity of the condition rather than any data collection limitation. Furthermore, the use of a standardized surgical technique by only two experienced surgeons throughout the entire study period reduces variability. Additionally, all transplants involved adult recipients and whole-organ grafts, and aspirin prophylaxis was uniformly administered; these are factors that help mitigate potential bias related to the extended timeframe.

## 5. Conclusions

Although the incidence of HAT in our cohort was low, its clinical consequences are severe and often necessitate surgical intervention or re-OLT. Risk factors such as an elevated donor-to-recipient body weight ratio, prolonged warm ischemia time, and the need for re-OLT were significantly associated with an increased HAT risk. Based on our findings, we strongly recommend the routine use of Doppler ultrasound in the immediate postoperative period, particularly within the first 10 days, in order to allow for the early detection of HAT, which is often asymptomatic. This protocol enabled timely interventions that preserved graft function in two-thirds of early HAT cases. Additionally, in patients with high-risk features, enhanced surveillance and early preventive measures, such as careful surgical technique and hemodynamic optimization, should be implemented. Adopting these strategies may help to reduce the incidence of HAT, improve graft survival, and minimize the need for re-OLT.

## Figures and Tables

**Table 1:** Variables considered in statistical analysis.

Donor-Related Variables	Recipient-Related Variables	Intraoperative Variables
Age Sex (M vs. F) Cause of death: Traumatic brain injury Intracerebral hemorrhage Ischemic stroke Post-anoxic encephalopathy Brain edema Gunshot wound CMV seropositivity (Yes/No) Anti-HBcAg antibody positivity (Yes/No) ICU stays Cardiac arrests (Yes/No) Hypotensive episodes (Yes/No) Blood transfusions (Yes/No) Administration of inotropes (Yes/No) Graft steatosis (Yes/No) Macrovesicular steatosis Microvesicular steatosis Body weight ratio (BWR, recipient weight/donor weight) Positive microbiological test on organ preservation fluid (Yes/No)	Age Sex (M vs. F) BMI CMV seropositivity (Yes/No) CMV-negative recipient with CMV-positive donor graft (Yes/No) Indication for transplantation: HCC HCV-related cirrhosis HBV-related cirrhosis HBV-HDV-related cirrhosis HCV recurrence Fulminant acute hepatitis Primary non-function (PNF) Delayed non-function (DNF) Exotoxin-related cirrhosis Wilson’s disease Cryptogenic cirrhosis Autoimmune hepatitis Primary biliary cirrhosis Secondary biliary cirrhosis Primary sclerosing cholangitis Polycystic liver disease Hemangioendothelioma Adenomatosis Metabolic cirrhosis Drug-induced cirrhosis Other Biological MELD score Child–Pugh score Pre-OLT HCC treatment (Yes/No)	Standard OLT (elective setting) (Yes/No) Re-OLT (Yes/No) Combined liver-kidney transplantation (Yes/No) Total ischemia time (CIT) Warm ischemia time (WIT) Use of arterial conduits (Yes/No) Arterial reconstructions at back table (Yes/No) Ligation of GDA at OLT (Yes/No) Ligation of SA at OLT (Yes/No) Ligation of both GDA and SA at OLT (Yes/No) More than one arterial anastomosis (Yes/No) Rescue cavo-cavostomy (Yes/No) Piggyback technique (Yes/No) Biliary reconstruction using choledochocholedochal or hepaticocholedochal anastomosis (Yes/No) Placement of a Kehr tube (Yes/No)

**Table 2:** Baseline donor characteristics.

Variable	*n* (%)
Number of OLTs	532
Media age (IQR)	57.5 (42–72) years
Gender	M 285 (53.6%) F 247 (46.4%)
Median Body Mass Index (BMI, IQR)	24.97 (23.04–27.14) Kg/cm^2^
Cause of death	
Traumatic brain injury	133 (25%)
Cerebral hemorrhage	304 (57.1%)
Ischemic stroke	48 (9.0%)
Post-anoxic encephalopathy	42 (7.9%)
Cerebral edema	3 (0.5%)
Gunshot wound	2 (0.3%)
CMV+	436 (81.9%)
Anti-HBcAb-positive	95 (17.8%)
Intensive Care Unit stay	4.17 (3.9%)
Cardiac arrests	76 (14.2%)
Hypotensive episodes	146 (28.3%)
Blood derivatives used	115 (21.6%)
Use of inotropes	437 (81.2%)
Graft steatosis	245 (46.0%)
Macrovescicular steatosis >30%	181 (34.0%)
Median body weight ratio (IQR)	1 (0.87–1.18)
Median graft weight (IQR)	1378 (1166–1581) g
Preservation fluid positive	149 (28.1%)

**Table 3:** Baseline recipient characteristics.

Variable	*n* (%)
OLTs	532 (492 patients)
Median age (IQR)	54 (48–60) years
Gender	M 405 (82.3%) F 87 (17.7%)
Median BMI (IQR)	23.9 (22.6–26.8) Kg/cm^2^
CMV+	446 (90.6%)
dCMV+ to rCMV-	45 (8.4%)
Both donor and recipient CMV−	14 (2.6%)
Median MELD score (IQR)	16 (11–22)
Child–Pugh score	
A (5–6)	152 (28.5%)
B (7–9)	233 (43.8%)
C (10–14)	114 (21.4%)
Pre-OLT HCC treatment	80/202 (39.6%)

**Table 4:** Indications for OLT.

Indications for OLT	*n* (%)
Hepatocellular carcinoma (HCC)	202 (37.0%)
Cirrhosis related to HCV infection (PHCC)	175 (32.8%)
Cirrhosis related to HBV infection (PHBC)	76 (14.2%)
Cirrhosis related to HBV-HDV co-infection (PHBC + PHDC)	36 (6.7%)
Recurrent viral hepatitis	10 (1.8%)
Fulminant hepatitis (FUHE)	16 (3.0%)
Primary non-function (PNF)	16 (3.0%)
Delayed non-function (DNF)	7 (0.1%)
Toxic cirrhosis (e.g., alcohol-related) (ALCI)	250 (46.9%)
Wilson’s disease	18 (3.3%)
Cryptogenic cirrhosis	9 (1.6%)
Autoimmune liver cirrhosis	19 (3.5%)
Primary biliary cirrhosis	10 (1.8%)
Secondary biliary cirrhosis	2 (0.3%)
Primary sclerosing cholangitis	4 (0.7%)
Polycystic liver disease	4 (0.7%)
Hemangioendothelioma	1 (0.1%)
Adenomatosis	1 (0.1%)
Metabolic cirrhosis	24 (4.5%)
Drug-induced cirrhosis	1 (0.1%)
Others	9 (1.6%)

**Table 5:** Intra-operative factors.

Variable	*n* (%)
Types of OLT	
Standard	435 (81.7%)
Early	47 (8.8%)
Urgent	50 (9.3%)
Re-OLT	37 (6.9%)
Combined transplantation (liver–kidney)	8 (1.5%)
Median time of cold ischemia (IQR)	400 (350.5–462) min
Median warm ischemia time (IQR)	30 (25–37) min
Types of arterial anastomosis	
Standard	470 (88.3%)
Non-standard	62 (11.6%)
Arterial conduits	4 (0.7%)
Back table anastomosis	149 (28.0%)
Arterial ligation	
Gastroduodenal artery	135 (25.3%)
Splenic artery	13 (2.4%)
Gastroduodenal and splenic artery	300 (56.3%)
Multiple vascular anastomosis	14 (2.6%)
Choledochocholedochal anastomosis	290 (54.5%)

**Table 6:** Types of arterial reconstructions during the OLTs and the relative HAT rate.

Types of Arterial Reconstruction	HAT Rate	*n* (%)
Standard Reconstruction		
dPHA–rPHA	6 (6.5%)	92 (17.2%)
dCHA–rPHA	2 (1.3%)	155 (29.1%)
dPHA–rCHA	1 (3.0%)	33 (6.2%)
dCHA–rCHA	1 (1.4%)	73 (13.7%)
dPHA–rRHA	1 (2.6%)	38 (7.1%)
dCHA–rRHA	-	45 (8.4%)
Other Reconstruction	2 (5.7%)	35 (6.6%)

PHA: proper hepatic artery. CHA: common hepatic artery. RHA: right hepatic artery. r: recipient. d: donors.

**Table 8:** Factors influencing the occurrence of HAT.

Risk Factors for the Occurrence of HAT
Donor-related factors
Body weight ratio (BWR, recipient weight/donor weight)
Intraoperative factors
Need for re-OLT due to graft failure
Warm ischemia time
Need for rescue cavo-cavostomy

## Data Availability

The database used for the analysis was coded and anonymous.
